# Transverse dentoalveolar changes of mandibular canine and premolar regions after lip bumper therapy: a retrospective CBCT study

**DOI:** 10.3389/froh.2025.1605132

**Published:** 2025-07-04

**Authors:** Jiahui Li, Normand S. Boucher, Chun-Hsi Chung, Shalin Shah, Chenshuang Li

**Affiliations:** ^1^ Department of Orthodontics, School of Dental Medicine, University of Pennsylvania, Philadelphia, PA, United States; ^2^ Private Practice, Princeton Junction, NJ, United States

**Keywords:** cone beam computed tomography (CBCT), expansion, lip bumper, alveolar bone, orthodontics

## Abstract

**Objectives:**

Lip bumpers (LB) treatment has been used to expand the mandibular arch during mixed dentition. The aim of this study is to evaluate the effects of LB on the mandibular transverse changes in the canine and premolar regions using CBCT.

**Materials and methods:**

This retrospective study utilized pre- (T1) and post-treatment (T2) CBCT images from the children who were treated either with rapid maxillary expander (RME) alone (RME group) or with RME and lip bumpers (RME + LB group) for interceptive orthodontic treatment. The T1 (pre-interceptive orthodontic treatment evaluation) and T2 (pre-comprehensive orthodontic treatment evaluation) CBCT images from the children who did not go through the interceptive orthodontic treatment were used as control. The CBCT images were oriented according to the occlusal plane and the three-dimensional superimposition on the mandible of T1 and T2 images was performed in the Dolphin 3D software, followed by a series of dental and alveolar linear and angular measurements. Only the mandibular canine and premolar regions with solid primary teeth that showed root structure below the furcation bilaterally at T1 and permanent teeth fully erupted in occlusion bilaterally at T2 were included. The intergroup comparisons were performed using the Mann–Whitney *U* test.

**Results:**

As the control group did not have a sufficient number of subjects after excluding the non-qualified regions, the following comparisons were only performed and reported between the RME group and the RME + LB group. RME + LB group (*n* = 30, 9.00 ± 0.86 years old at T1, 11.99 ± 0.59 years old at T2) showed significantly more bodily buccal movement of mandibular canines and premolars than the RME group (*n* = 25, 8.72 ± 0.88 years old at T1, 12.00 ± 0.96 years old at T2), but inter-mandibular buccal surface width increase was only observed in the second premolar region. In addition, the RME + LB groups showed less buccal alveolar bone thickness and height than the RME group in the mandibular canine and first premolar regions.

**Conclusion:**

LB significantly expanded the mandibular transverse dimension dentally, with permanent canine and premolars erupting more buccally. However, it does not increase the skeletal transverse dimension of the alveolar bone at the canine and first premolar regions. Further studies are needed to evaluate the long-term effects of LB.

## Introduction

Dentoskeletal transverse deficiency is one of the significant components in orthodontic analysis and diagnosis (1). In contrast to the maxilla, for which a significant number of studies have evaluated the efficiency of skeletal transverse expansion by separating the intermaxillary suture using various maxillary expander designs, there are limited reports on evaluating the skeletal expansion of the mandible as there is no midline suture in the mandibular body postnatally.

The lip bumper appliance has been used as one of the mandibular expansion methods, in either an active form, which is activated by expanding the archwire to be slightly wider than buccal tubes on the molars to achieve transverse expansion, or a passive form, which passively shields the lip and cheek musculature away from the mandibular dentition to allow the spontaneous expansion of the mandible (1). In the 1990s, Osborn et al. (2) examined the transverse effect of passive lip bumpers on the mandibular posterior dentition. They found that the change in muscular pressure of the cheek and tongue indirectly increased the inter-canine width by 1.99 mm, the inter-first premolar width by 2.5 mm, and the inter-second premolar width by 2.43 mm. This study was questioned since there was also a variety of maxillary appliances being used in combination with the LB in this study, and the maxillary expanders have been reported to affect the mandibular arch due to the “occlusion dragging effects” (3–6). However, the effects of a passive LB were further proven by studies from two different groups who evaluated subjects treated with passive LB without maxillary expansion, and reported 1.78–1.8 mm inter-canine width increase, 3.39 mm inter-first premolar width increase, and 1.83–2.58 mm inter-second premolar width increase in the mandibular arch at the dental cusps level (7, 8). As expected, the mandibular dental transverse increase in the canine, premolar, and molar regions was also reported with active LB, in which a more significant amount of expansion was observed in the canine and premolar regions than the passive LB (9, 10).

Despite the valuable insights from the aforementioned literature, the mandibular transverse dimension was analyzed utilizing dental models exclusively. Thus, existing literature predominantly focuses on the dental effect of LB on mandibular transverse change. The only report on the skeletal effects of LB was published in 2004, Vanarsdall et al. (11) evaluated posteroanterior (PA) cephalograms and reported a larger skeletal mandibular transverse increase at the antegonial notch (AG) level in subjects treated with RME + LB than in subjects only treated with braces. However, these findings were questioned due to the limitations of two-dimensional radiographic images, such as distortions, magnifications, and overlaps of structures (12). To further understand the treatment effects of LB in the transverse dimension of the mandible, especially for the subjects experiencing dentition transition during LB treatment, this current retrospective, longitudinal CBCT study aims to investigate the dentoalveolar transverse changes of mandibular canines and premolars associated with LB.

## Material and methods

### Subjects included in the current study

The protocol of this study was approved by the University of Pennsylvania institution review board (protocol # 852263, approved on October 10th, 2022). This retrospective study utilized the same pool of patients treated in the same private practices by the same clinicians as the previous study (13), which included treatment samples started over ten years ago in two separate clinics. Thus, the appliance design and activation protocol, as well as the CBCT acquirement process are the same as described in the study by Orr *et al*. (13). In brief, three groups were utilized for this study (Table 1):

**Table 1:** Demographic information of involved subjects.

Measurements		Control	RME	RME + LB	*P* value of ANOVA test	*P* value of *t*-test (RME vs. RME + LB)
All subjects included in the current study						
Subject Number		10	25	30	–	–
Age	T1 (years)	9.17 ± 1.31	8.72 ± 0.88	9.00 ± 0.86	0.3622	0.2410
	T2 (years)	12.24 ± 1.21	12.00 ± 0.96	11.99 ± 0.59	0.6964	0.9564
	T1 to T2 Time (years)	3.07 ± 1.19	3.28 ± 0.82	2.99 ± 0.64	0.4109	0.1450
Skeletal Pattern	SNA (degrees)	80.04 ± 2.20	79.90 ± 3.19	80.66 ± 3.75	0.6878	0.4289
	SNB (degrees)	77.66 ± 2.88	76.43 ± 3.76	77.60 ± 3.14	0.3898	0.2142
	ANB (degrees)	2.40 ± 1.85	3.46 ± 2.05	3.24 ± 1.87	0.3452	0.6745
	Wits (mm)	−1.83 ± 2.89	−0.56 ± 3.41	−0.79 ± 2.31	0.4906	0.7710
Subjects included in the measurements of canine region						
Subject number		7	21	24	–	–
Age	T1 (years)	8.51 ± 0.82	8.56 ± 0.84	8.88 ± 0.91	0.4059	0.2394
	T2 (years)	11.74 ± 0.72	12.04 ± 1.02	11.94 ± 0.61	0.6992	0.6911
	T1 to T2 Time (years)	3.23 ± 1.35	3.48 ± 0.75	3.06 ± 0.65	0.2404	0.0543
Skeletal Pattern	SNA (degrees)	80.14 ± 2.19	79.85 ± 2.87	80.37 ± 3.83	0.8685	0.6112
	SNB (degrees)	77.13 ± 2.50	76.69 ± 3.49	77.18 ± 2.74	0.8552	0.5987
	ANB (degrees)	3.03 ± 1.70	3.16 ± 2.02	3.42 ± 1.86	0.8505	0.6563
	Wits (mm)	−0.84 ± 2.19	−1.06 ± 3.45	−0.95 ± 2.34	0.9824	0.8987
Subjects included in the measurements of first premolar region						
Subject number		7	23	28	–	–
Age	T1 (years)	9.13 ± 1.18	8.68 ± 0.85	8.95 ± 0.87	0.4097	0.2728
	T2 (years)	12.16 ± 0.84	12.07 ± 0.92	11.99 ± 0.61	0.8615	0.7293
	T1 to T2 Time (years)	3.02 ± 1.18	3.39 ± 0.78	3.04 ± 0.63	0.2426	0.0854
Skeletal Pattern	SNA (degrees)	79.93 ± 1.57	80.36 ± 2.89	80.38 ± 3.70	0.9427	0.9821
	SNB (degrees)	78.13 ± 2.44	77.10 ± 3.06	77.16 ± 2.64	0.6773	0.9475
	ANB (degrees)	1.80 ± 1.61	3.25 ± 1.95	3.42 ± 1.78	0.1164	0.7460
	Wits (mm)	−2.61 ± 2.87	−1.11 ± 2.93	−0.72 ± 2.36	0.2506	0.6033
Subjects included in the measurements of second premolar region						
Subject number		5	16	24	–	–
Age	T1 (years)	9.24 ± 1.42	8.85 ± 1.00	8.87 ± 0.89	0.7191	0.9344
	T2 (years)	12.57 ± 1.26	11.89 ± 1.01	12.00 ± 0.59	0.2936	0.6672
	T1 to T2 Time (years)	3.32 ± 1.00	3.04 ± 0.84	3.13 ± 0.64	0.7704	0.7231
Skeletal Pattern	SNA (degrees)	78.54 ± 1.49	79.35 ± 3.42	80.18 ± 3.97	0.5863	0.5013
	SNB (degrees)	77.00 ± 2.76	75.69 ± 3.98	77.28 ± 3.23	0.3677	0.1732
	ANB (degrees)	1.56 ± 1.35	3.66 ± 1.93	3.13 ± 2.01	0.1176	0.4143
	Wits (mm)	−2.32 ± 1.85	−0.48 ± 3.30	−0.94 ± 2.28	0.4086	0.6070

The subject number represented the number of children involved in each group. Data are presented as mean ± standard deviation.

Control group (28 subjects in the database, 12 males and 16 females, 8.99 ± 1.62 years old at T1, 11.24 ± 1.83 years old at T2): patients who had CBCTs taken as pre-orthodontic treatment records (T1), but did not undergo treatment. On average, three years later, they returned for a second CBCT (T2) as pre-orthodontic records.

RME group (30 subjects in the database, 17 males and 13 females, 8.76 ± 0.98 years old at T1, 12.10 ± 1.00 years old at T2): patients treated with a bonded Haas expander with posterior occlusal coverage (14) and no treatment in the lower arch. CBCTs were taken as pre-orthodontic records before expansion (T1), and pre-orthodontic records before fixed appliance therapy (T2). Subjects had 11 mm expansion (one complete round of expansion with 11 mm jackscrew) with 0.5 mm daily activation.

RME + LB group (35 subjects in the database, 15 males and 20 females, 9.10 ± 0.85 years old at T1, 12.03 ± 0.56 years old at T2): patients who received a bonded Haas expander with posterior occlusal coverage for the maxilla (14) and active lip bumper (Dentsply GAC international, NY, USA) (7) therapy for the mandible. CBCTs were obtained before treatment (T1), and pre-orthodontic records before fixed appliance therapy (T2). All patients had the maxillary expansion of 8.5 mm with a bonded Haas expander (one complete round of expansion with a single 8.5 mm jackscrew) with 0.5 mm activation per day. The treatment length of LB ranged from 1.16 years to 4.28 years, with a median treatment time of 1.94 years. The LB was activated transversely by expanding the wire facially to 1 mm wider than the buccal tubes on the mandibular first molars during each appointment until proper buccal-lingual inclination was achieved on the mandibular first molars based on the clinical judgment by the same clinician.

To accurately present the primary and permanent tooth buccal-lingual inclination, for each subject, only the regions with primary teeth showing root structure below the furcation bilaterally at T1 and permanent teeth fully erupted in occlusion bilaterally at T2 were included (Table 1).

### CBCT image processing

**Figure 1:**
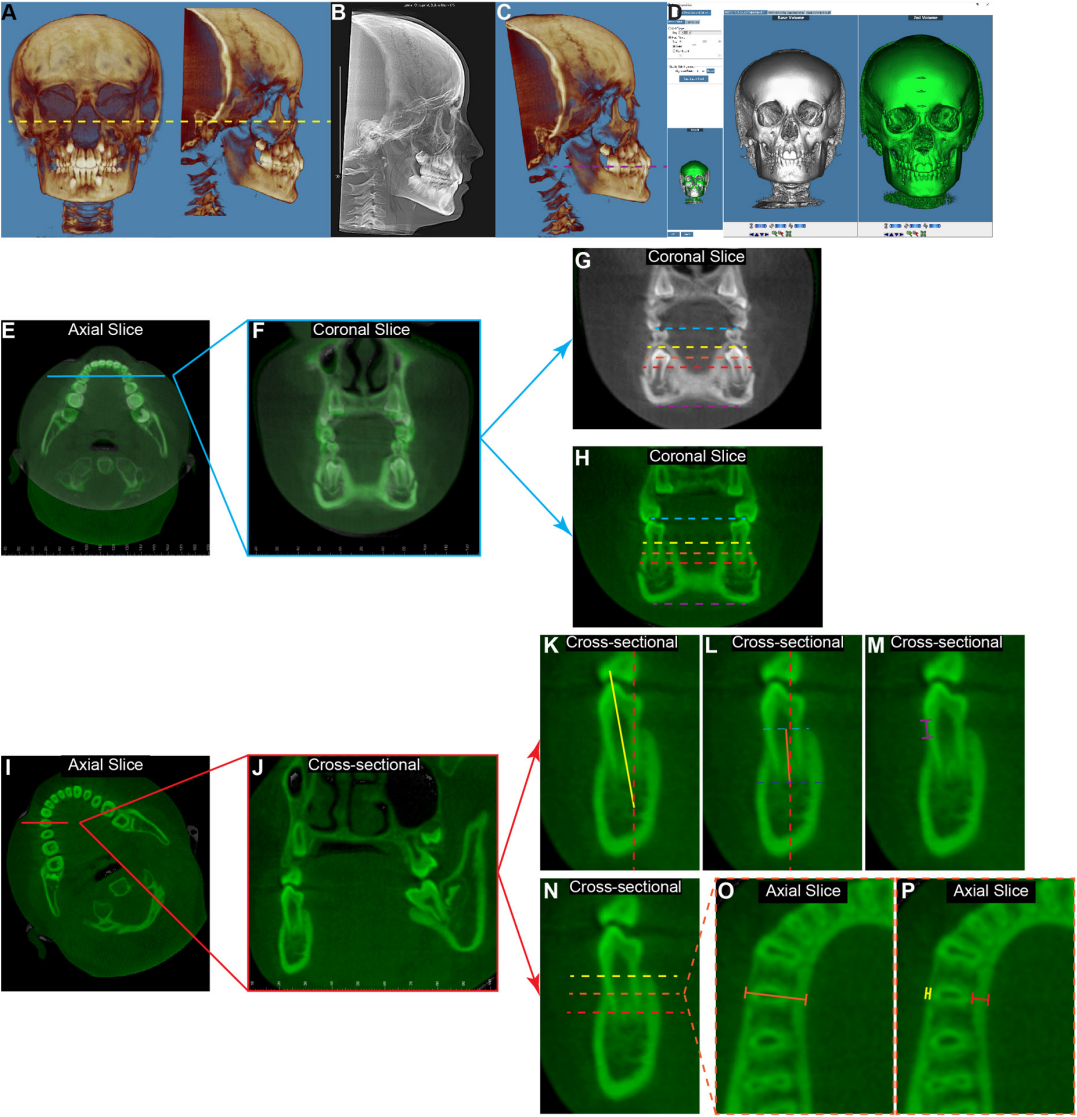
The diagram of measurement protocol for the mandibular first premolar region. **(A)** The initial orientation of T1 3D reconstructed image was set according to the Frankfurt plane to have the left and right orbitale points, as well as the right porion point on the same horizontal plane (yellow dashed line). **(B)** The lateral cephalometric x-ray was extracted for the cephalometric tracing. **(C)** The CBCT images were then re-orientated to have the occlusal plane (purple dashed line) as the horizontal plane on the side view. **(D)** By utilizing the “overlay superimposition” function in the Dolphin 3D image superimposition, the T1 image (white) and the T2 image (green) were superimposed based on the anterior region of the mandible. Using the measurements in the mandibular first premolar region as an example, on the axial view bisecting the mandibular dentition **(E)**, the coronal slice **(F)** was defined as the slice going through the meso-distal midpoint of the mandibular first premolars. Then the inter-primary first molar width at T1 [**(G)**, blue dashed line] and the inter-permanent first premolar width at T2 [**(H)**, blue dashed line] were measured by connecting the buccal cusps of the corresponding teeth. The buccal-buccal surface widths were measured at the same coronal sides of T1 **(G)** and T2 **(H)** at the levels of 2 mm (yellow dashed line), 5 mm (orange dashed line), and 9 mm (red dashed line) below the cementoenamel junction (CEJ) of the mandibular right first premolar (LR4) at T2. The inter-mandibular lower border widths (purple dashed line) were measured as connecting the most inferior points of the left and right sides. For the tooth and alveolar bone measurements of LR4, the axial view of the T2 image **(I)** was oriented to have the horizontal line parallel with the buccal-lingual long axial of LR4. Thus, the coronal slice **(J)** would present as bisecting the LR4. On this coronal view, the LR4 inclination **(K)** was measured as the angulation formed by the long axis of the tooth (yellow solid line) and the true vertical line (red dashed line). The alveolar ridge inclination **(L)** was measured as the angulation formed by the line (orange solid line) connecting the midpoint of the alveolar ridge at the alveolar crest level (light blue dashed line) and the root apex level (dark blue dashed line), and the true vertical line (red dashed line). For both angulation measurements, the value was recorded as positive if the object was lingually inclined, and it was recorded as negative if the object was buccally inclined. The buccal alveolar bone level **(M)** was measured as the distance between the CEJ of the tooth and the tip of the alveolar crest. **(N)** At the levels 2 mm (yellow dashed line), 5 mm (orange dashed line), and 9 mm (red dashed line) below the LR4 CEJ, the total alveolar ridge thickness [**(O)**, orange solid line], the buccal alveolar bone thickness [**(P)**, yellow solid line], and the lingual alveolar bone thickness [**(P)**, red solid line] were measured at the corresponding axial slice.

CBCT DICOM files were imported into Dolphin 3D software (Dolphin Imaging; version 11.95 Premium, Chatsworth, CA) for visualization and processing. Frankfort Horizontal Plane was used as the reference for the initial orientation of the CBCT (Figure 1A) to eliminate any head malposition in the orientation of yaw, roll, or pitch (13). The non-magnified lateral cephalometric x-rays were extracted from the T1 CBCT images for cephalometric tracing (Figure 1B) (15). Then the T1 CBCT was reoriented by using the occlusal plane as the horizontal plane (Figure 1C) to allow coronal slices being perpendicular with the occlusal plane, and the T2 CBCTs were superimposed on T1 CBCTs by using the voxel-based superimposition method adapted from the 2D ABO mandibular superimposition (Figure 1D) (16, 17). Linear and angular measurements were performed in the canine, first premolar, and second premolar regions (Figures 1E–P).

Interdental width: by using the cusp tip as the landmark for primary and permanent canines, and the buccal cusp tip as the landmark for primary molars and permanent premolars, the distance between primary teeth at T1 (Figure 1G, blue dashed line) and between permanent teeth at T2 (Figure 1H, blue dashed line) were measured.

Mandibular width: at each tooth region, the coronal slice for transverse measurements was defined as the slice that goes through the meso-distal midpoint of the mandibular tooth on both sides at T2 (Figures 1E,F). The mandibular transverse widths were measured at the alveolar levels 2 mm, 5 mm, and 9 mm below the cementoenamel junction (CEJ) of each mandibular right permanent tooth at T2 (Figure 1H, yellow, orange, and red dashed lines) and at the mandibular lower border level (Figure 1H, purple dashed line). To ensure that these skeletal measurements were being taken at the same anterior-posterior position at T1 and T2, the same slice from the superimposition over T2 was utilized for T1 (Figure 1G).

Tooth buccal-lingual inclination: the T2 CBCT image was oriented to have the coronal slice bisecting the corresponding tooth buccal-lingually (Figures 1I,J). Then, on the coronal slice, the long axis of the tooth was identified as a line connecting the midpoint of the apex and the cusp tip of the tooth. The tooth inclination was defined as the angle between this long axis of the tooth and the true vertical line (Figure 1K). The buccal inclination of the crown was deemed to be negative, and the lingual inclination of the crown was deemed to be positive.

Alveolar ridge inclination: the alveolar ridge inclination was measured on the same coronal slice as the tooth inclination. The long axis of the alveolar ridge was identified as a line connecting the midpoint of the alveolar ridge at the alveolar crest level and the root apex level. The alveolar ridge inclination was defined as the angle between this long axis and the true vertical (Figure 1L). The buccal inclination of the alveolar ridge was deemed to be negative, and the lingual inclination of the alveolar ridge was deemed to be positive.

Buccal alveolar bone level: on the same coronal slice that measures tooth inclination, the distance between the CEJ of the tooth at the buccal surface and the tip of the alveolar crest was measured to represent the buccal alveolar bone level of the corresponding permanent tooth at T2 (Figure 1M).

Alveolar ridge thickness and alveolar bone thickness: the evaluations of alveolar thickness were performed at the levels 2 mm, 5 mm, and 9 mm below the CEJ of the corresponding permanent tooth at T2 (Figure 1N). On the same coronal slice that measures tooth inclination, the alveolar ridge thickness is defined as the distance between the mandibular buccal surface and the lingual surface (Figure 1O). In addition, the buccal alveolar bone thickness and lingual alveolar bone thickness were also measured (Figure 1P).

### Statistical analysis

In this study, all the measurements were performed by one examiner in a blinded fashion. To check the consistency and reliability of the current measuring method, the CBCT files of five subjects were randomly selected and measured again one month after the first round of measurement. The intra-class correlation coefficient (ICC) of the measurements were calculated and paired *t*-test was implemented utilizing the IBM SPSS software (Statistical Package for Social Sciences version 26.0, Chicago, IL, USA).

The sample size was determined based on power analysis with *α* = 0.05, 80% power, and a Cohen's d of 1.2, which represents a ‘very large' effect size (18). A minimum of 13 samples per group was needed to ensure an adequate sample size for showing statistical differences.

Since the current study only focused on the region with solid primary teeth presented bilaterally at T1 and permanent teeth fully erupted in occlusion bilaterally at T2, the subjects of each group that can be included in each tooth region varied (Table 1). After excluding the non-qualified regions, the control group did not have a sufficient number of subjects. Thus, statistical comparisons were only performed between the RME and RME + LB groups. The control group's data were still included in the current study for reference due to the difficulty of obtaining longitudinal CBCT images to observe the mandibular transverse growth and development without orthodontic intervention.

The demographic data were all presented by mean ± standard deviation. Independent *t*-test was performed for demographic data comparison. For all other measurements, the Shapiro–Wilk normality test was conducted by GraphPad Prism (Version 8.2.1, GraphPad Software, San Diego, USA). Since some data did not pass the normal distribution test, data were presented as median [minimum, maximum], and a non-parametric Mann–Whitney *U*-test was used for statistical comparison. For each tooth region, measurements of both left and right sides were performed. Non-parametric Mann–Whitney *U*-test revealed that there was no statistically significant difference on each measurement between left and right sides. Thus, the data of both sides were combined for further analysis. For all data presented, *P* < 0.05 was used as a statistically significant difference.

## Results

### Comparisons of patients’ demographic information

After applying inclusive criteria as described above, there were 10 subjects included in the control group (6 males and 4 females, 9.17 ± 1.31 years old at T1, 12.24 ± 1.21 years old at T2), 25 subjects in the RME group (14 males and 11 female, 8.72 ± 0.88 years old at T1, 12.00 ± 0.96 years old at T2), and 30 subjects in the RME + LB group (13 males and 17 female, 9.00 ± 0.86 years old at T1, 11.99 ± 0.59 years old at T2) (Table 1).

For the total sample population included in this study, there was no difference in the subjects’ age (age at T1, age at T2, and T1 to T2 time), maxillary sagittal position (SNA), mandibular sagittal position (SNB), and maxillo-mandibular sagittal relation (ANB, Wits). In addition, for each region, there were also no statistically significant differences between groups in all the demographic information measured (Table 1).

Among the measured variables, the ICC ranged from 0.901 (for mandibular canine inclination) to 1.000 (for Buccal alveolar bone thickness 2 mm below CEJ at canine region). In addition, no statistically significant difference was found by the paired *t*-test. Thus, there is high consistency and reliability of the current measurement protocol.

### LB led to dental expansion in the canine and premolar regions by bodily buccal movement of the teeth

**Figure 2:**
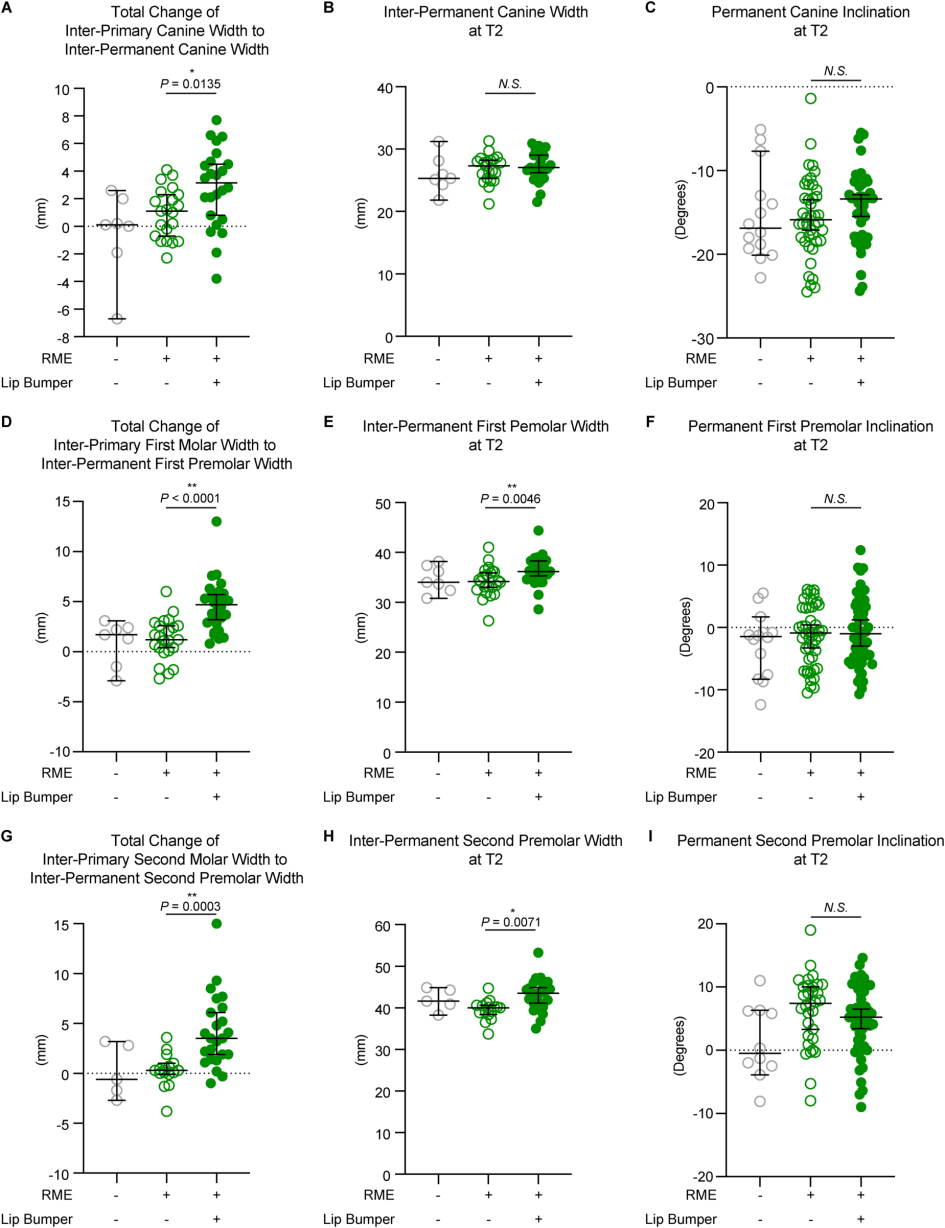
The dental measurements of all three groups. The total changes between T1 and T2 at the canine **(A)**, first premolar **(D)**, and second premolar **(G)** regions demonstrated a significant increase of inter-dental width in the RME + LB group compared to the RME group. The inter-dental width at T2 at the canine **(B)**, first premolar **(E)**, and second premolar **(H)** regions demonstrated significantly wider arch at the premolars’ region in the RME + LB group compared to the RME group. The tooth buccolingual inclination measurements at T2 at the canine **(C)**, first premolar **(F)**, and second premolar **(I)** regions demonstrated no statistically significant difference between the RME group and the RME + LB group. All the data are presented as raw data overlayed with median ± 95% confidence interval. N.S, no statistically significant; *: *P* < 0.05; **: *P* < 0.005.

When comparing changes from inter-primary tooth width to inter-permanent tooth width, the RME + LB group is associated with a statistically significant increase in canine (RME 1.10 mm [−2.30 mm, 4.10 mm] vs. RME + LB 3.15 mm [−3.80 mm, 7.70 mm), first premolar (RME 1.20 mm [−2.70 mm, 6.00 mm] vs. RME + LB 4.70 mm [0.80 mm, 13.00 mm), and second premolar (RME 0.30 mm [−3.80 mm, 3.60 mm] vs. RME + LB 3.50 mm [−1.00 mm, 15.00 mm) regions compared to the RME group (Figure 2). In addition, the RME + LB group also presented with significantly wider inter-premolar widths at T2 than the RME group (at the first premolar region: RME 0.30 mm [−3.80 mm, 3.60 mm] vs. RME + LB 3.50 mm [−1.00 mm, 15.00 mm]; at the second premolar region: RME 40.05 mm [33.70 mm, 44.70 mm] vs. RME + LB 43.50 mm [35.00 mm, 53.30 mm) (Figure 2).

We then evaluated permanent tooth inclination to determine if the increases in inter-tooth widths in the RME + LB group were due to buccal tipping or bodily movement, and no statistically significant difference was found between the RME and the RME + LB group for all three teeth inclination at T2 (canine: RME 1.10° [−2.30°, 4.10°] vs. RME + LB 3.15° [−3.80°, 7.70°]; first premolar: RME −0.90° [−10.50°, 6.10°] vs. RME + LB −1.00° [−10.70°, 12.40°]; second premolar: RME 7.40° [−8.00°, 19.00°] vs. RME + LB 5.20° [−9.00°, 14.60°) (Figure 2). Thus, LB did not cause buccal tipping of the canine and premolars.

### LB caused different responses in the mandibular transverse change at canine and premolar regions

**Figure 3:**
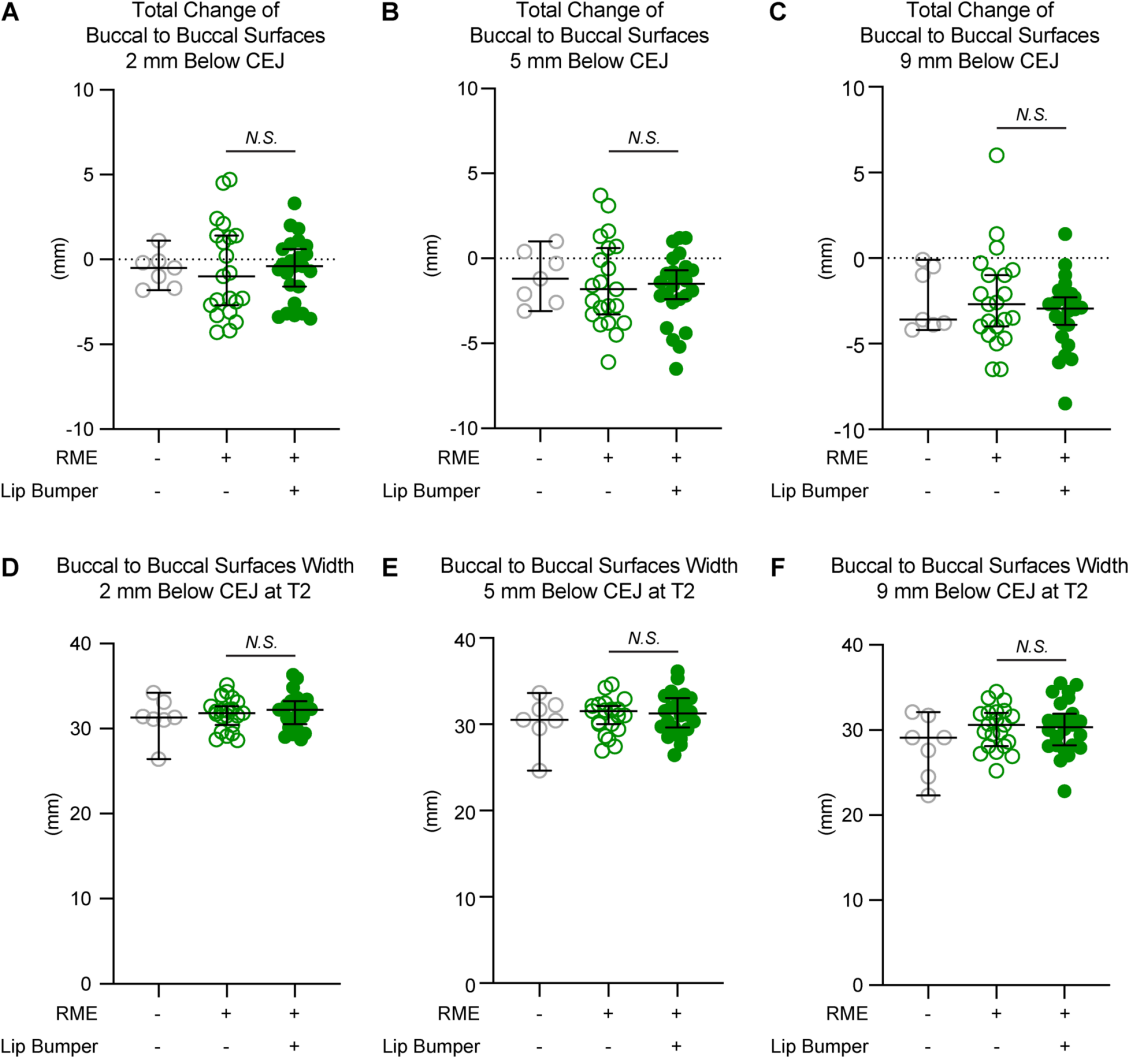
The mandibular buccal surface-buccal surface distance measurements at the canine region. The total changes between T1 and T2 at the level 2 mm **(A)**, 5 mm **(B)**, and 9 mm **(C)** below the cementoenamel junction (CEJ) of the mandibular right permanent canine at T2 demonstrated no statistically significant difference between the RME group and the RME + LB group. The mandibular buccal surface-buccal surface distance at T2 at the level 2 mm **(D)**, 5 mm **(E)**, and 9 mm **(F)** below the CEJ of the mandibular right permanent canine at T2 demonstrated no statistically significant difference between the RME group and the RME + LB group. All the data are presented as raw data overlayed with median ± 95% confidence interval. N.S, no statistically significant.

**Figure 4:**
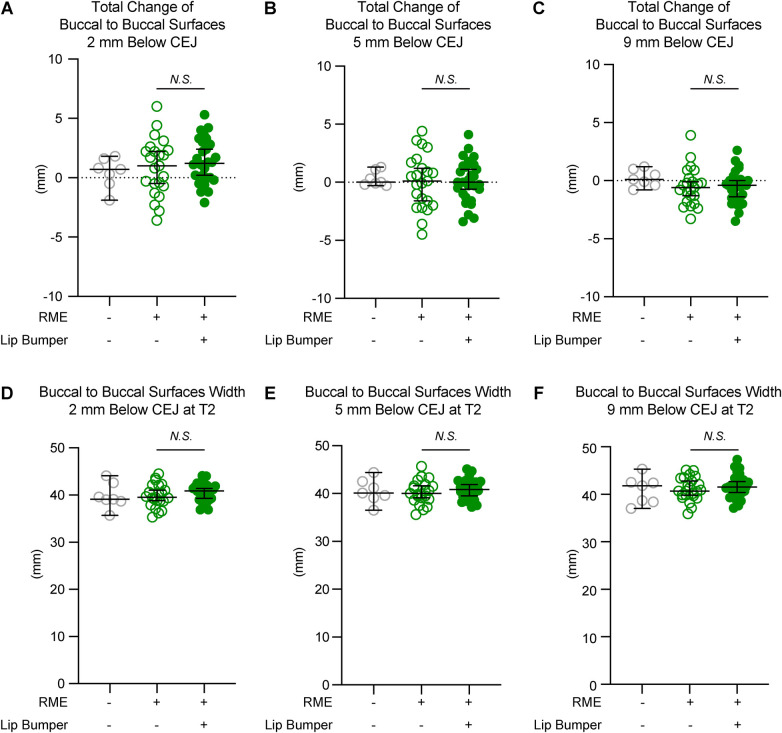
The mandibular buccal surface-buccal surface distance measurements at the first premolar region. The total changes between T1 and T2 at the level 2 mm **(A)**, 5 mm **(B)**, and 9 mm **(C)** below the cementoenamel junction (CEJ) of the mandibular right first premolar at T2 demonstrated no statistically significant difference between the RME group and the RME + LB group. The mandibular buccal surface-buccal surface distance at T2 at the level 2 mm **(D)**, 5 mm **(E)**, and 9 mm **(F)** below the CEJ of the mandibular right first premolar at T2 demonstrated no statistically significant difference between the RME group and the RME + LB group. All the data are presented as raw data overlayed with median ± 95% confidence interval. N.S., no statistically significant.

**Figure 5:**
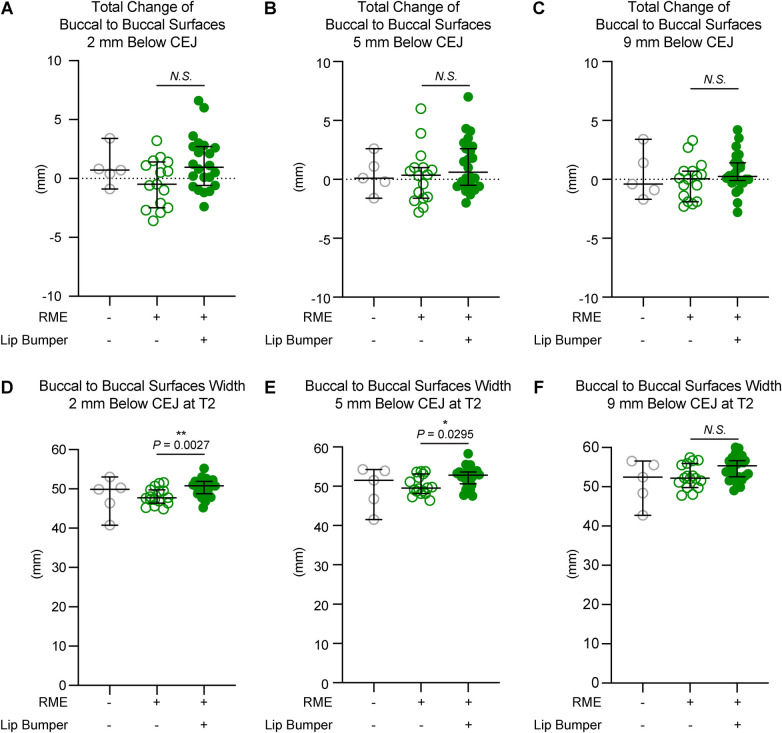
The mandibular buccal surface-buccal surface distance measurements at the second premolar region. The total changes between T1 and T2 at the level 2 mm **(A)**, 5 mm **(B)**, and 9 mm **(C)** below the cementoenamel junction (CEJ) of the mandibular right first premolar at T2 demonstrated no statistically significant difference between the RME group and the RME + LB group. The RME + LB group has significantly wider mandibular buccal surface-buccal surface distance at T2 than the RME group at the levels 2 mm **(D)** and 5 mm **(E)** below the CEJ of the mandibular right second premolar, but not at the level 9 mm **(F)** below the CEJ of the mandibular right second premolar. All the data are presented as raw data overlayed with median ± 95% confidence interval. N.S., no statistically significant. *: *P* < 0.05; **: *P* < 0.005.

**Figure 6:**
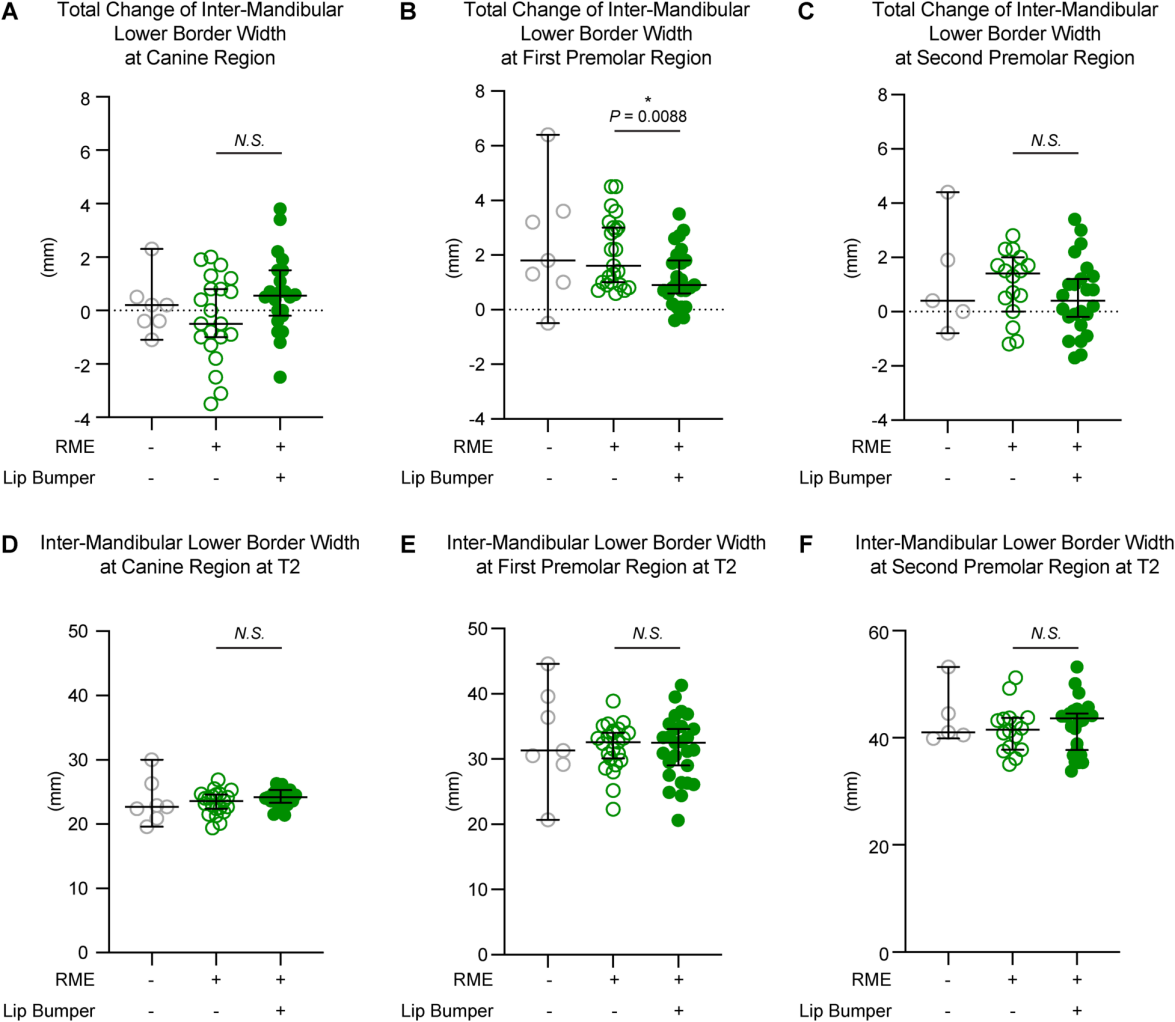
The inter-mandibular lower border distance measurements of all three groups. The total changes between T1 and T2 demonstrated no significant difference between the RME and the RME + LB groups at the canine **(A)** and second premolar **(C)** regions, but a significant difference between groups at the first premolar **(B)** region. The inter-mandibular lower border distance measurements at T2 at the canine **(D)**, first premolar **(E)**, and second premolar **(F)** regions demonstrated no statistically significant difference between the RME group and the RME + LB group. All the data are presented as raw data overlayed with median ± 95% confidence interval. N.S., no statistically significant; *: *P* < 0.05; **: *P* < 0.005.

To further evaluate if LB could cause skeletal expansion, the mandibular transverse changes from T1 to T2, and the mandibular transverse width at T2, were evaluated at the levels 2 mm, 5 mm, and 9 mm below the CEJ of the corresponding permanent teeth (Figures 3–5), as well as at the mandibular lower border (Figure 6).

The RME and RME + LB groups had similar inter-mandibular buccal surface widths at the alveolar level in the canine and first premolar regions (Figuress 3,4). However, in the second premolar region, a significantly wider mandible was observed in the RME + LB group at levels 2 mm and 5 mm below CEJ (at the level 2 mm below CEJ: RME 47.70 mm [44.90 mm, 51.60 mm] vs. RME + LB 50.80 mm [45.20 mm, 55.20 mm]; at the level 5 mm below CEJ: RME 49.50 mm [46.40 mm, 53.80 mm] vs. RME + LB 52.85 mm [47.50 mm, 58.30 mm) (Figure 5).

At the base bone level, no significant difference was observed between the RME and RME + LB groups in all three teeth regions (at the canine region: RME 23.60 mm [19.40 mm, 26.90 mm] vs. RME + LB 24.20 mm [21.40 mm, 26.30 mm]; at the first premolar region: RME 32.60 mm [22.30 mm, 38.90 mm] vs. RME + LB 32.50 mm [20.60 mm, 41.30 mm]; at the second premolar region: RME 41.55 mm [35.00 mm, 51.20 mm] vs. RME + LB 43.65 mm [33.80 mm, 53.20 mm) (Figure 6).

### LB caused buccal alveolar bone loss of canines and first premolars

Since different amounts of expansions were observed at the dental and skeletal levels in the canine and premolar regions, we further evaluate the alveolar bone morphology around each permanent tooth at T2.

First of all, the total alveolar ridge thickness and the alveolar bone inclination were similar in the RME and RME + LB groups in all three regions, except the alveolar bone was more lingually inclined in the first premolar region in the RME + LB group (Table 2).

**Table 2:** Alveolar ridge thickness and inclination at T2.

Tooth	Measurements	Control	RME	RME + LB	*P* value of Mann–Whitney *U* test RME vs. RME + LB
Canine	Sample number	14	42	48	–
	2 mm Below CEJ (mm)	10.15 [7.40, 12.30]	9.70 [7.60, 13.60]	9.70 [7.10, 12.40]	0.4845
	5 mm Below CEJ (mm)	10.50 [6.80, 12.80]	9.50 [7.40, 13.30]	9.40 [6.70, 12.70]	0.4285
	9 mm Below CEJ (mm)	10.95 [6.50, 13.40]	9.75 [6.60, 13.90]	9.95 [6.90, 12.90]	0.6019
	Inclination (°)	−12.05 [−18.10, −3.80]	−10.45 [−17.20, 8.80]	−8.05 [−26.30, −1.70]	0.4287
First Premolar	Sample number	14	46	56	–
	2 mm Below CEJ (mm)	9.55 [7.70, 13.30]	9.50 [7.30, 13.00]	9.10 [6.50, 12.20]	0.3503
	5 mm Below CEJ (mm)	9.90 [6.90, 12.60]	10.05 [7.20, 13.90]	10.20 [7.00, 12.10]	0.9453
	9 mm Below CEJ (mm)	11.05 [8.00, 13.40]	10.70 [8.10, 14.20]	10.55 [8.20, 13.50]	0.6236
	Inclination (°)	−4.65 [−12.30, 3.10]	−0.15 [−10.20, 12.30]	3.50 [−18.10, 16.40]	0.0498
Second Premolar	Sample number	10	32	48	–
	2 mm Below CEJ (mm)	9.00 [7.70, 13.30]	9.75 [7.70, 12.60]	10.15 [6.90, 14.00]	0.4858
	5 mm Below CEJ (mm)	9.75 [7.50, 14.40]	11.30 [8.80, 14.00]	11.55 [7.90, 14.50]	0.4617
	9 mm Below CEJ (mm)	10.90 [9.00, 13.60]	11.80 [9.60, 14.30]	12.25 [9.30, 14.50]	0.4982
	Inclination (°)	12.85 [−7.50, 18.20]	12.60 [4.20, 26.00]	14.25 [−8.50, 27.80]	0.7939

The sample number represented the number of teeth involved in each group. Data are presented as median [minimum, maximum]. CEJ, cementoenamel junction. *P < 0.05; **P < 0.005.

Second, when evaluating the buccal and lingual alveolar bone thickness (Tables 3, 4), the RME + LB group had significantly thinner buccal alveolar bone at the region 5 mm below the canine CEJ (Table 3), buccal alveolar bone at the region 2 mm below the first premolar CEJ (Table 3), and lingual alveolar bone at the region 9 mm below the second premolar CEJ (Table 4) than the RME group. The thinning of the buccal alveolar bone in the canine and first premolar regions in the RME + LB group was further proven by the increased distance between CEJ and the alveolar crest, as shown in Table 3.

**Table 3:** Buccal alveolar bone thickness and height at T2.

Tooth	Measurements	Control	RME	RME + LB	*P* value of Mann–Whitney *U* test RME vs. RME + LB
Canine	Sample number	14	42	48	–
	Thickness at 2 mm Below CEJ (mm)	0.00 [0.00, 0.80]	0.00 [0.00, 0.30]	0.00 [0.00, 0.80]	0.5967
	Thickness at 5 mm Below CEJ (mm)	0.70 [0.00, 0.90]	0.35 [0.00, 1.10]	0.00 [0.00, 1.30]	0.0488*
	Thickness at 9 mm Below CEJ (mm)	1.20 [0.60, 1.70]	1.10 [0.60, 2.00]	1.05 [0.20, 1.90]	0.0768
	Alveolar bone crest level (mm)	3.60 [1.00, 6.00]	4.25 [1.00, 7.60]	5.20 [1.60, 7.90]	0.0455*
First Premolar	Sample number	14	46	56	–
	Thickness at 2 mm Below CEJ (mm)	0.65 [0.00, 1.10]	0.00 [0.00, 0.80]	0.00 [0.00, 0.80]	0.0062*
	Thickness at 5 mm Below CEJ (mm)	0.70 [0.00, 1.30]	0.70 [0.00, 1.40]	0.70 [0.00, 1.70]	0.4915
	Thickness at 9 mm Below CEJ (mm)	1.30 [0.80, 1.70]	1.10 [0.70, 2.10]	1.25 [0.60, 2.20]	0.5035
	Alveolar bone crest level (mm)	1.40 [0.00, 4.60]	1.95 [0.00, 4.60]	2.90 [0.00, 6.10]	0.0134*
Second Premolar	Sample number	10	32	48	–
	Thickness at 2 mm Below CEJ (mm)	0.75 [0.00, 1.20]	0.55 [0.00, 1.30]	0.50 [0.00, 1.40]	0.7135
	Thickness at 5 mm Below CEJ (mm)	1.15 [0.00, 2.00]	1.20 [0.30, 1.90]	1.30 [0.40, 2.50]	0.2859
	Thickness at 9 mm Below CEJ (mm)	1.30 [0.80, 2.30]	1.60 [0.90, 2.30]	1.70 [0.90, 2.60]	0.1904
	Alveolar bone crest level (mm)	1.40 [0.00, 5.40]	0.70 [0.00, 4.30]	1.20 [0.00, 3.90]	0.1212

The sample number represented the number of teeth involved in each group. Data are presented as median [minimum, maximum]. CEJ: cementoenamel junction. *P < 0.05; **P < 0.005.

**Table 4:** Lingual alveolar bone thickness at T2.

Tooth	Measurements	Control	RME	RME + LB	*P* value of Mann–Whitney *U* test RME vs. RME + LB
Canine	Sample number	14	42	48	–
	2 mm Below CEJ (mm)	1.35 [0.00, 1.90]	1.40 [0.20, 3.60]	1.40 [0.00, 2.70]	0.7394
	5 mm Below CEJ (mm)	1.45 [0.00, 2.30]	1.45 [0.50, 2.80]	1.45 [0.50, 2.70]	0.8228
	9 mm Below CEJ (mm)	1.80 [0.70, 2.20]	1.60 [0.40, 2.50]	1.60 [0.80, 2.60]	0.5566
First Premolar	Sample number	14	46	56	–
	2 mm Below CEJ (mm)	1.65 [0.00, 2.80]	1.60 [0.30, 3.40]	1.40 [0.40, 2.60]	0.1604
	5 mm Below CEJ (mm)	1.70 [0.50, 2.70]	1.90 [0.70, 3.40]	1.90 [0.90, 3.20]	0.3653
	9 mm Below CEJ (mm)	1.95 [1.50, 2.70]	1.95 [0.80, 3.00]	1.90 [1.10, 2.90]	0.2750
Second Premolar	Sample number	10	32	48	–
	2 mm Below CEJ (mm)	0.80 [0.00, 2.40]	1.15 [0.60, 2.00]	1.10 [0.60, 2.20]	0.9941
	5 mm Below CEJ (mm)	1.45 [0.60, 2.70]	1.80 [1.00, 2.70]	1.80 [0.60, 2.80]	0.1149
	9 mm Below CEJ (mm)	1.90 [0.90, 2.40]	2.10 [1.10, 2.60]	1.80 [1.00, 2.50]	0.0072*

The sample number represented the number of teeth involved in each group. Data are presented as median [minimum, maximum]. CEJ, cementoenamel junction. *P < 0.05; **P < 0.005.

## Discussion

With the long history of lip bumper usage in orthodontics, the evaluations on treatment effects of lip bumper were predominantly performed on dental casts, which could only provide information on interdental width changes. For the skeletal evaluations, Vanarsdall et al. (11) reported a larger skeletal mandibular transverse increase at the antegonial notch (AG) level in subjects treated with RME + LB than in subjects only treated with braces. While this study was based on posteroanterior cephalograms evaluation, the findings from Vanarsdall et al. (11) could not be validated by a current cone-beam computed tomography (CBCT) based longitudinal study (13). In fact, in the CBCT study, Orr et al. reported that active LB increased the inter-molar width by uprighting the mandibular first molars, and the alveolar bone around the mandibular first molars responded to the dental changes. However, there was a decrease in the alveolar bone buccolingual thickness at the molar furcation level, even though the buccal alveolar bone thickness in the first molar was maintained (13). In addition, no mandibular width increase was observed at the basal bone level at the first molar region (13).

It is worth noting that although a similar amount of expansion was observed in the canine, premolar, and molar regions in LB treatment from previous studies on dental casts, the underlying mechanisms are entirely different. The first molars are the teeth that carry the expansion force from the active LB appliance so that the dentoalveolar response may be closer to orthodontic tooth movement. The canine and premolar regions, especially for the subjects who had primary teeth at the beginning of LB treatment and had permanent teeth at the end of LB treatment, were responding to the musculature force changes, which may alter the permanent teeth eruption path. This hypothesis is partially proved by Moin et al., who reported bodily movement of mandibular canine and premolars in LB expansion by evaluating dental casts (Moin and Bishara), which contrasts the buccal tipping of mandibular first molars by evaluating CBCT (13). Although both tooth eruption and orthodontic tooth movement involve alveolar bone remodeling, the processes have fundamental differences (19). Thus, the dentoskeletal changes in the mandibular first molar region in response to LB therapy could not be directly applied to the canine and premolar regions.

The current study demonstrated that different from the increase in the inter-molar width by uprighting in the mandibular first permanent molar region (13), LB resulted in the bodily buccal movement of canines and premolars during the dentition transition, but inter-mandibular buccal surfaces increase was only observed in the second premolar region. The discrepancy between dental and alveolar changes led to reductions in buccal alveolar bone thickness and height in the canine and first premolar regions, as opposed to the alveolar bone remodeling around the mandibular first molars in response to the dental changes (13).

At the dental level, the differences between the RME group and the RME + LB groups on the increase from T1 (primary dentition) to T2 (permanent dentition) were 2.05 mm expansion in the canine region, 3.50 mm expansion in the first premolar region, and 4.40 mm in the second premolar region. Since both groups had the same type of RME and both groups had similar ages at T1 and T2, the differences observed here can be considered as the pure effects of active LB. The amounts of inter-canine and inter-premolar expansions observed in the current study are similar to those reported previously based on the dental casts evaluation (2, 7–10, 20), which further proves the reliability of the current study. By comparing the dental inclination at T2 between RME and RME + LB groups, the current study also demonstrated that the dental transverse increase was achieved by bodily movement of the canine and premolars, which is the same as the results reported by Moin et al (10). based on the evaluation of dental casts.

The most significant advantage of using CBCT in evaluating the transverse dimension is the accessibility to the skeletal structures. As the tooth bud's location at T1 would significantly alter the evaluation of the alveolar ridge, the assessment on the alveolar bone in the current study was only limited to T2. In the current study, despite the significant amount of expansion in the canine and first premolar regions, the alveolar bone remodeling didn’t follow the dental changes since there was no significant difference in mandibular buccal-buccal surface distance, alveolar ridge thickness, as well as in the alveolar ridge inclination between the RME and RME + LB groups at T2, to the exception of the more lingually inclined alveolar ridge in the first premolar region in the RME + LB group. These changes completely differ from the same parameters in the molar regions, where the alveolar bone would remodel to the new dental position (13). The discrepancy between the dental and alveolar changes in the canine and first premolar regions led to the buccal alveolar bone loss. A previous study has demonstrated that when evaluating the buccal alveolar bone thickness in the mandibular canine to second molar regions, the thinnest bone was found in both the canine and first premolar regions (21). Although no orthodontic force was applied to the canines and first premolars, the current study demonstrated that passive expansion in the mandibular canine and first premolar regions could compromise the periodontal tissue. However, the long-term effects of LB on the periodontal status in these regions need to be further evaluated. In addition, whether LB in phase I treatment provides similar effects compared to pure dental arch expansion from arch wires or clear aligners in phase II treatment should be investigated in future studies.

Interestingly, the RME + LB group showed a wider mandibular body than the RME group in the second premolar region at T2. Although more dental expansion was observed in the second premolar region than the canine and first premolar regions in the RME + LB group, there was no buccal alveolar bone thinning or loss in the second premolar region. This might be because the mandibular second premolar is close to the external oblique ridge of the mandible. The oblique ridge functions as the attachment of the buccinator muscle, which presses the check against the teeth (22). The LB may stretch the buccinator muscle or alter the force transmission from the muscle to the bony attachment, which in turn, causes bone adaptation (23, 24). However, this hypothesis needs further validation, as no study is available on evaluating the muscle tone changes caused by LB.

Nevertheless, there are some limitations of the current study that must be addressed. First, due to the nature of the retrospective study, we could not randomly allocate the involved subjects in each group. Thus, the subjects in each treatment group may also present the treatment selection bias from the clinicians. Second, due to the number of subjects involved in the current study, we could not stratify the subjects based on the skeletal vertical or sagittal patterns. A recent study showed that hyperdivergent and hypodivergent subjects have different maxillary dentoskeletal changes in response to rapid maxillary expander therapy in early mixed dentition (25), which may be due to the various sizes and forces of the masseter muscle (26–28). Thus, whether subjects with different skeletal patterns respond to LB therapy differently needs to be evaluated. Thirdly, there is limitation of evaluating the periodontal bone using CBCT. The voxel size of all the CBCT images involved in the current study is 0.3 mm, so when a subject is smaller than 0.3 mm, it would be difficult to be identified on the CBCT images (29–31). Thus, the exact values of alveolar bone thickness and of the distance between the CEJ and alveolar bone crest cannot be taken directly from this study. However, with the high accuracy and consistency of the measurement method utilized in the current study, the conclusion of overall less buccal alveolar bone in the RME + LB group than in the RME group is solid and cannot be overlooked. Last but not least, the current study only includes the time points of pre-interceptive orthodontic treatment (T1) and pre-comprehensive orthodontic treatment (T2). While the dentoalveolar structure goes through constant changes due to growth and development as well as due to comprehensive orthodontic management, Whether the dentoalveolar effects observed in this treatment phase could last after the comprehensive orthodontic treatment is unknown. Further studies are needed to evaluate the long-term effects of LB.

## Conclusion

In conclusion, this retrospective CBCT study demonstrates that LB therapy produces significant transverse dental expansion in the mandibular arch at the end of interception orthodontic management. However, LB cannot achieve mandibular skeletal expansion in the canine and first premolar regions, and leads to buccal alveolar bone loss in these regions. In the second premolar region, skeletal expansion was observed at the alveolar level, but the underlying mechanism is unknown. Further studies are needed to evaluate the long-term dental and periodontal status of patients treated with LB.

## Data Availability

The original contributions presented in the study are included in the article/Supplementary Material, further inquiries can be directed to the corresponding author.
